# Vision transformer-based ordinal model for semi-quantitative synovitis grading on musculoskeletal ultrasound: a retrospective study with internal and external validation

**DOI:** 10.1080/07853890.2026.2738281

**Published:** 2026-10-01

**Authors:** Xin Liu, Jing Li

**Affiliations:** Department of Ultrasound, Second Hospital of Tianjin Medical University, Tianjin, China

**Keywords:** Deep learning, musculoskeletal ultrasound, synovitis, Vision Transformer

## Abstract

**Background:**

To develop and validate a Vision Transformer-based model for semi-quantitative grading of synovitis on musculoskeletal ultrasound and to assess its potential clinical utility. The study also examined whether transformer-based modeling offers advantages over conventional CNNs in ordinal ultrasound grading.

**Patients:**

This retrospective diagnostic study included 312 patients, 624 joints, and 1,248 ultrasound images, which were divided at the patient level into training, validation, internal test, and external test cohorts. A Swin Transformer–based ordinal grading model was trained on gray-scale and power Doppler ultrasound data and compared with CNN baselines using internal validation, external testing, stratified subgroup analysis, interpretability assessment, and a reader-assistance evaluation.

**Results:**

The Vision Transformer achieved the best overall performance, with an internal-test accuracy of 0.809, macro F1 score of 0.806, weighted kappa of 0.842, and AUC of 0.929, while evaluation on an external test set, curated from a non-overlapping period at the same institution, remained acceptable with an accuracy of 0.771 and AUC of 0.904. Most errors occurred between adjacent grades, multimodal GSUS plus PDUS input outperformed single-modality models, and AI assistance improved junior-reader agreement and efficiency.

**Conclusions:**

This preliminary study confirms that Vision Transformer can achieve accurate and clinically applicable synovitis grading on musculoskeletal ultrasound, showing better robustness than traditional CNNs. This method may help standardize and streamline ultrasound evaluation in clinical practice and multicenter studies.

## KEY MESSAGES

1. A Vision Transformer-based model outperforms conventional CNNs for semi-quantitative synovitis grading on musculoskeletal ultrasound;
2. Multimodal input combining gray-scale and power Doppler ultrasound improves grading performance;
3. AI assistance enhances junior readers’ agreement and efficiency, supporting more standardized and efficient clinical ultrasound assessment.

## 1. Introduction

Synovitis is an important imaging feature of joint inflammation and a clinically meaningful marker of disease activity in musculoskeletal disorders [1], including osteoarthritis and inflammatory arthropathies. In osteoarthritis in particular, synovial inflammation has been associated with pain severity, functional limitation, and structural progression [2], underscoring the need for reliable assessment in both research and clinical care. Musculoskeletal ultrasound is well suited to this purpose because it is accessible, dynamic, and sensitive to synovial hypertrophy, joint effusion, and vascular activity [2]. Despite these advantages, ultrasound-based grading remains dependent on operator expertise and visual interpretation, and semi-quantitative scoring is still affected by inter-observer variability [3], especially in borderline cases and across different imaging settings.

Recent advances in artificial intelligence have created new opportunities for automated analysis of musculoskeletal ultrasound, yet robust grading of synovitis remains challenging [4]. Conventional convolutional neural networks(CNN) have shown value in medical image classification [5], but their performance may be constrained in fine-grained grading tasks where subtle differences in morphology, echotexture [6], and spatial context determine score assignment [7]. Ultrasound images are particularly difficult because of speckle noise, indistinct lesion boundaries [8], and substantial heterogeneity across joints, patients, and acquisition protocols [9]. These characteristics suggest that synovitis grading requires a model that can integrate localized inflammatory findings with broader anatomical context rather than relying only on local texture patterns.

Vision transformer architectures offer a potentially suitable framework for this problem because self-attention enables the modeling of long-range relationships and global contextual information within an image [10]. To our knowledge, few prior studies have evaluated transformer-based architectures specifically for ordinal semi-quantitative synovitis grading with external validation and multimodal ultrasound integration.

On this basis, the present study investigates whether a Vision Transformer-based approach can provide accurate and clinically relevant grading of synovitis on musculoskeletal ultrasound. We hypothesized that, compared to traditional convolutional neural networks, a Vision Transformer-based approach would: (1) better capture global and diffuse textural patterns of synovitis *via* self-attention, (2) improve discrimination between adjacent semi-quantitative grades. The aim is to develop and validate a model for semi-quantitative synovitis grading and to assess its potential as a tool for improving standardization, reproducibility, and scalability in ultrasound-based joint assessment.

## 2. Materials and methods

### 2.1. Study design and data source

This retrospective diagnostic study included consecutive patients who underwent musculoskeletal ultrasound for symptomatic peripheral joints at The Second Hospital of Tianjin Medical University between April 2025 and March 2026 [11]. Inclusion criteria were: (1) age ≥ 18 years; (2) symptomatic peripheral joint (metacarpophalangeal, proximal interphalangeal, knee or wrist) examined by ultrasound; (3) standardized grayscale and power Doppler images available; and (4) complete clinical records accessible [12]. Exclusion criteria were: (1) poor image quality precluding confident interpretation (e.g. severe attenuation, acoustic shadowing); (2) major postoperative distortion or joint replacement; (3) marked artifacts obscuring the synovial recess; and (4) duplicate same‑visit follow‑up scans [13]. The cohort included osteoarthritis, rheumatoid arthritis, and undifferentiated inflammatory arthritis. The final dataset comprised 312 patients, 624 joints, and 1,248 images. Baseline characteristics were summarized at both the patient and image levels [14]. Mean age was 56.8 ± 13.4 years, and 214 of 312 patients were women. Osteoarthritis, rheumatoid arthritis, and other inflammatory arthritides accounted for 138, 124, and 50 patients, respectively. A formal sample size calculation was not performed prior to the study, as there is no established method for sample size determination in deep learning‑based ordinal classification of ultrasound images. Instead, we followed commonly recommended guidelines in medical imaging AI research: (1) at least 50 samples per class for classification tasks with moderate complexity, (2) patient‑level separation to avoid data leakage, and (3) inclusion of an external validation set. Our final dataset of 312 patients (1,248 images) provided a minimum of 200 images per semi‑quantitative grade (range 249–372), which exceeds the widely cited heuristic of 50–100 samples per class for deep learning models. Moreover, the sample size is comparable to or larger than those used in previous studies applying transformer architectures to musculoskeletal ultrasound [15,16]. Therefore, the sample size is considered adequate for the exploratory aims of this study. Ultrasound examinations were performed using three commercially available high-frequency ultrasound systems from different manufacturers, including Mindray Resona A20、Aplio i800、GE fortis. To preserve the independence of external evaluation and avoid potential bias related to specific vendors, device information was anonymized during model development and statistical analysis. All systems used linear-array transducers with nominal frequencies 18 and 15 MHz.

The distribution of demographic, diagnostic, and imaging variables across the development and test cohorts is shown in Table 1.

**Table 1.** Baseline characteristics of the study population and imaging dataset.

Variable	Overall *n* = 312	Development *n* = 218	Internal test *n* = 47	External test *n* = 47
Age, years	56.8 ± 13.4	56.5 ± 13.1	57.4 ± 14.0	57.6 ± 14.2
Female sex, *n* (%)	214 (68.6)	150 (68.8)	31 (66.0)	33 (70.2)
Osteoarthritis, *n* (%)	138 (44.2)	97 (44.5)	21 (44.7)	20 (42.6)
Rheumatoid arthritis, *n* (%)	124 (39.7)	85 (39.0)	19 (40.4)	20 (42.6)
Other inflammatory arthritis, *n* (%)	50 (16.0)	36 (16.5)	7 (14.9)	7 (14.9)
Examined joints, *n*	624	436	94	94
Images or analyzed views, *n*	1,248	872	188	188
Knee joints, *n* (%)	248 (39.7)	176 (40.4)	36 (38.3)	36 (38.3)
Wrist joints, *n* (%)	176 (28.2)	120 (27.5)	28 (29.8)	28 (29.8)
MCP joints, *n* (%)	124 (19.9)	88 (20.2)	18 (19.1)	18 (19.1)
PIP joints, *n* (%)	76 (12.2)	52 (11.9)	12 (12.8)	12 (12.8)
Gray-scale only exams, *n* (%)	86 (27.6)	58 (26.6)	14 (29.8)	14 (29.8)
Gray-scale plus power Doppler exams, *n* (%)	226 (72.4)	160 (73.4)	33 (70.2)	33 (70.2)
Transducer frequency, MHz	15.0 (12.0–18.0)	15.0 (12.0–18.0)	15.0 (12.0–18.0)	14.0 (12.0–18.0)
Vendor A, *n* (%)	148 (47.4)	111 (50.9)	19 (40.4)	18 (38.3)
Vendor B, *n* (%)	104 (33.3)	69 (31.7)	17 (36.2)	18 (38.3)
Vendor C, *n* (%)	60 (19.2)	38 (17.4)	11 (23.4)	11 (23.4)

### 2.2. Reference standard and annotation procedure

The reference standard was expert semi-quantitative synovitis grading on a 0–3 scale.Synovitis was graded according to the EULAR-OMERACT consensus-based semi‑quantitative scoring system [17]^.^ Although originally validated predominantly in rheumatoid arthritis, this morphological and vascular semiquantitative scoring system has been broadly applied to other inflammatory and degenerative conditions, including osteoarthritis and undifferentiated arthritis [18,19], and has been validated across multiple joint sites (e.g. wrist, knee, metacarpophalangeal, and proximal interphalangeal joints) in prior literature, supporting its applicability to our heterogeneous cohort and multi-joint data [20,21].^.^ Two senior musculoskeletal sonographers independently reviewed all images after atlas-based calibration [22], and disagreements were resolved by a third expert [23]. Inter-reader agreement before adjudication was substantial, with a quadratic-weighted Cohen’s kappa of 0.81.

All images were anonymized and independently graded by two senior sonographers who were blinded to clinical data, with disagreements resolved by a third blinded expert; readers were unavoidably aware of the joint site for anatomical localization. Across the full dataset, grades 0, 1, 2, and 3 were assigned to 286, 372, 341, and 249 images, respectively, showing moderate but clinically realistic imbalance. Grade distribution remained comparable across cohorts, supporting valid internal evaluation of discrimination and calibration. Detailed synovitis grading data are listed in Table 2.

**Table 2.** Distribution of synovitis grades at the image level.

Synovitis grade	Overall *n* = 1,248	Development *n* = 872	Internal test *n* = 188	External test *n* = 188
Grade 0, *n* (%)	286 (22.9)	198 (22.7)	44 (23.4)	44 (23.4)
Grade 1, *n* (%)	372 (29.8)	262 (30.0)	55 (29.3)	55 (29.3)
Grade 2, *n* (%)	341 (27.3)	240 (27.5)	50 (26.6)	51 (27.1)
Grade 3, *n* (%)	249 (20.0)	172 (19.7)	39 (20.7)	38 (20.2)

### 2.3. Image preprocessing and dataset partition

All ultrasound images were processed through a uniform preprocessing pipeline before model development. Patient identifiers, machine annotations, and non-imaging borders were removed, and images were resized to 224 × 224 pixels with intensity normalization to a common grayscale range. Preprocessing also included speckle-aware normalization and mild contrast enhancement [24]. No vendor-specific preprocessing, histogram matching, or device-specific calibration was applied. This design was intended to evaluate the intrinsic robustness of the model to realistic inter-device variability rather than performance after device harmonization.

Dataset partitioning was performed strictly at the patient level [25]. All images from the same patient were assigned to one subset to prevent leakage between training and evaluation. An external test set was derived from non‑overlapping same‑institution data, using identical protocols and machines. It differed temporally, partially in operators, and was processed unseen after model finalization. This design permits assessment of robustness to temporal drift and operator dependence, serving as a rigorous intermediate step prior to multi-center validation [26]. In the main analysis, the 218 development patients were divided into 174 for training and 44 for validation, with stratification by diagnosis and grade distribution.

### 2.4. Transformer-based model development

The primary model was a Swin Transformer-Tiny architecture adapted for ordinal ultrasound grading [27]. This model was selected because its hierarchical window-based self-attention preserves local structural detail while enabling progressively broader contextual integration, which is advantageous for musculoskeletal ultrasound where subtle synovial abnormalities must be interpreted in relation to the surrounding joint recess. Each image was partitioned into non-overlapping patches and embedded into a latent feature space. For an input image $x\in R^{H\times W\times C}$, the patch embedding layer generated a token sequence $\left\{z_{1},z_{2},\ldots ,z_{N}\right\}$, where N is the number of patches. Self-attention within each transformer block was computed as

$$\text{Attention}(Q,K,V)=\text{Softmax}(\frac{QK^{⊤}}{\sqrt{d_{k}}})V$$

where Q, K, and V denote the query, key, and value matrices, and $d_{k}$ is the key dimension. The final representation was passed to an ordinal prediction head rather than a standard flat classifier, allowing the model to reflect the ordered structure of synovitis grades from 0 to 3.

The main learning objective combined weighted cross-entropy with an ordinal consistency term. Let y∈{0,1,2,3} denote the reference grade and ${\hat{p}}_{k}$ the predicted probability for class k. The weighted cross-entropy component was defined as $L_{\text{CE}}=-\sum_{k=0}^{3}\;w_{k}\;1(y=k)\text{log}({\hat{p}}_{k})$ where $w_{k}$ represents class-specific weights. To encourage respect for grade ordering, an additional ordinal loss was introduced based on cumulative probabilities $L_{\text{ord}}=\frac{1}{3}\sum_{t=0}^{2}\text{BCE}(c_{t},{\hat{c}}_{t})$, where $c_{t}=1(y>t)$ and ${\hat{c}}_{t}=P(\hat{y}>t)$. To incorporate the ordinal nature of synovitis severity, the training objective combined weighted cross-entropy loss and an ordinal consistency loss. The total loss was defined as: $\;L=L_{\text{CE}}+\lambda L_{\text{ord}}$. where $L_{\text{CE}}$ represents weighted cross-entropy loss for four-grade classification and $L_{\text{ord}}$ constrains the cumulative probability distribution across ordered grades. Specifically, the ordinal component encouraged monotonic cumulative probabilities, such that the probability of predicting a grade equal to or higher than a given threshold decreased progressively as the threshold increased. This design reduced inconsistent outputs, such as simultaneously assigning high probability to both mild and severe categories.

The weighting coefficient λ was set to 0.5 based on validation-set optimization and the clinical requirement to balance categorical discrimination with ordinal consistency. A lower weight provided insufficient constraint on grade ordering, whereas a higher weight excessively restricted class separation and reduced sensitivity for borderline grades. Therefore, λ=0.5 was selected as the optimal trade-off between classification accuracy and ordinal calibration.

The ordinal constraint influenced model behavior in two ways. First, it improved probability calibration by encouraging predicted distributions to follow the continuous progression of disease severity rather than independent class probabilities. Second, it refined decision boundaries by reducing abrupt transitions between distant grades and promoting more clinically plausible separation between adjacent categories. This was particularly relevant for distinguishing grades 1 and 2, where ultrasound findings often overlap.

ImageNet-pretrained weights were used for initialization. Training was performed with the AdamW optimizer, an initial learning rate of $1\times 10^{-4}$, weight decay of 0.05, cosine annealing, batch size of 32, and a maximum of 100 epochs. Early stopping was triggered when validation loss failed to improve for 12 consecutive epochs. Five-fold cross-validation within the development cohort was used to assess training stability and reduce dependence on a single split. In additional experiments, cropped lesion-focused inputs were concatenated with full-image features, and a multimodal branch incorporating diagnosis category and joint type was explored through late fusion.

### 2.5. Reader-assistance evaluation

To assess clinical utility, three junior musculoskeletal ultrasound readers (1–3 years of experience) independently graded test cases using standardized GSUS and PDUS images, without involvement in model development or label adjudication. A senior sonographer (>8 years) provided the reference standard. In the unassisted session, readers graded images without model input. After a 4-week washout, the same readers re-evaluated the cases with access to model-generated grade predictions and confidence outputs. All readers were blinded to expert labels, diagnoses, and model performance; case order was randomized, and readers made independent final decisions without altering images or acquisition parameters. The reader-assistance evaluation was conducted between March 2026 and April 2026, following the completion of model development and internal validation.

## 3. Results

### 3.1. Cohort characteristics and reference reliability

A total of 312 patients, 624 joints, and 1,248 ultrasound images were analyzed. The dataset was split into training, validation, internal test, and external test subsets(same-institution dataset with temporal separation), with balanced diagnosis, joint distribution, imaging-platform characteristics, and grade composition across cohorts. More than 70% of examinations in both test sets included combined gray-scale and power Doppler ultrasound. Reference labels showed good reproducibility, with a quadratic-weighted Cohen’s kappa of 0.81 before adjudication [28]. Agreement was highest for grades 0 and 3, while most disagreements occurred between grades 1 and 2. Detailed cohort characteristics and grade distribution are summarized in Tables 3 and 4.

**Table 3.** Cohort composition.

Subset	Patients	Joints	Images	OA, *n* (%)	RA, *n* (%)	Other arthritis, *n* (%)	Knee, *n* (%)	Wrist, *n* (%)	Hand joints, *n* (%)	Vendor A/B/C
**Training**	174	348	696	77 (44.3)	68 (39.1)	29 (16.7)	140 (40.2)	96 (27.6)	112 (32.2)	89/56/29
**Validation**	44	88	176	20 (45.5)	17 (38.6)	7 (15.9)	36 (40.9)	24 (27.3)	28 (31.8)	22/13/9
**Internal test**	47	94	188	21 (44.7)	19 (40.4)	7 (14.9)	36 (38.3)	28 (29.8)	30 (31.9)	19/17/11
**External test**	47	94	188	20 (42.6)	20 (42.6)	7 (14.9)	36 (38.3)	28 (29.8)	30 (31.9)	18/18/11

**Table 4.** Grade distribution.

Grade	Training *n* (%)	Validation *n* (%)	Internal test *n* (%)	External test *n* (%)
**0**	157 (22.6)	41 (23.3)	44 (23.4)	44 (23.4)
**1**	210 (30.2)	52 (29.5)	55 (29.3)	55 (29.3)
**2**	193 (27.7)	47 (26.7)	50 (26.6)	51 (27.1)
**3**	136 (19.5)	36 (20.5)	39 (20.7)	38 (20.2)

### 3.2. Overall model performance

The vision Transformer showed the best overall performance in the internal test set, with an accuracy of 0.809, a macro F1 score of 0.806, a weighted kappa of 0.842, and an AUC of 0.929. It outperformed ResNet-50, which achieved an accuracy of 0.734, a macro F1 score of 0.726, a weighted kappa of 0.768, and an AUC of 0.881, while DenseNet-121 and EfficientNet-B0 showed intermediate results [29]. Most Vision Transformer errors occurred between adjacent grades, whereas CNN baselines showed more frequent confusion between grades 1 and 2 and between grades 2 and 3, which are clinically important boundaries in semi-quantitative assessment [30].

Performance remained stable in external validation, where the Vision Transformer achieved an accuracy of 0.771, a macro F1 score of 0.768, a weighted kappa of 0.812, and an AUC of 0.904. Although these values were slightly lower than those of the internal test set, the decline was smaller than that of the CNN baselines, indicating greater robustness to acquisition variability and dataset heterogeneity [31]. The ordinal formulation further improved consistency [32], reducing mean absolute error from 0.287 to 0.223 and increasing within-one-grade accuracy from 0.920 to 0.957 compared with the standard multiclass version. Detailed results are presented in Figure 1.

**Figure 1.**
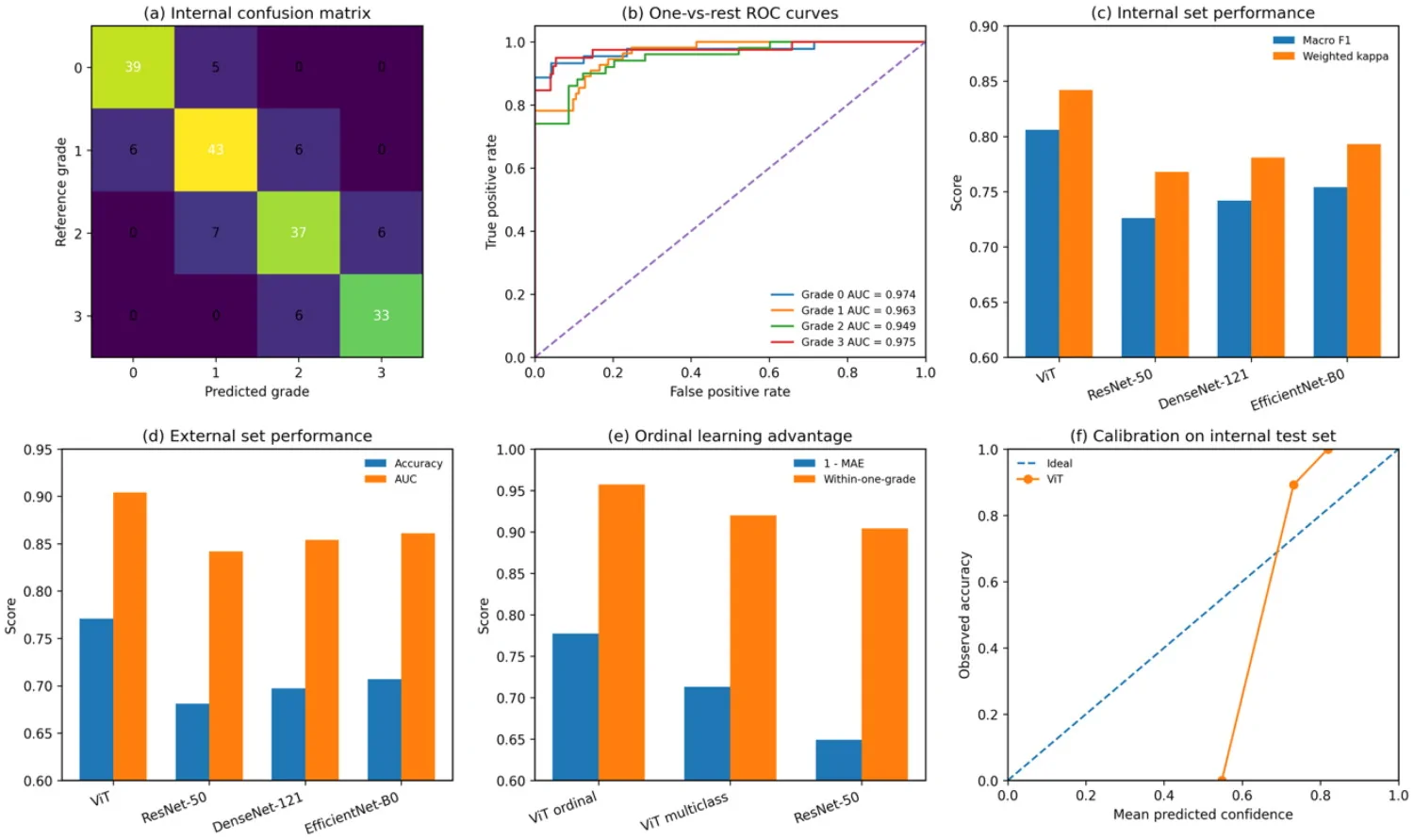
**Performance of the Vision Transformer for synovitis grading on musculoskeletal ultrasound.** (a) Confusion matrix of the Vision Transformer on the internal test set. (b) One-vs-rest receiver operating characteristic curves for grades 0 to 3 on the internal test set. (c) Comparison of macro F1 score and weighted kappa. (d) Comparison of accuracy and AUC across the same models in the external test set. (e) Advantage of ordinal learning. (f) Calibration plot of the Vision Transformer on the internal test set.

### 3.3. Stratified performance across joint sites

Stratified analysis showed modest variation across joint sites, with the best performance in the knee and slightly lower performance in the wrist and small joints. Macro F1 scores were 0.828 in the knee, 0.769 in metacarpophalangeal joints, and 0.742 in proximal interphalangeal joints, while the Vision Transformer remained superior to the CNN baseline across all sites [33]. Most disagreements were limited to adjacent grades, supporting stable ordinal discrimination. The model performed best in larger joints with clearer recess morphology, while maintaining acceptable consistency in small-joint ultrasound; see Figure 2 for detailed results.

**Figure 2.**
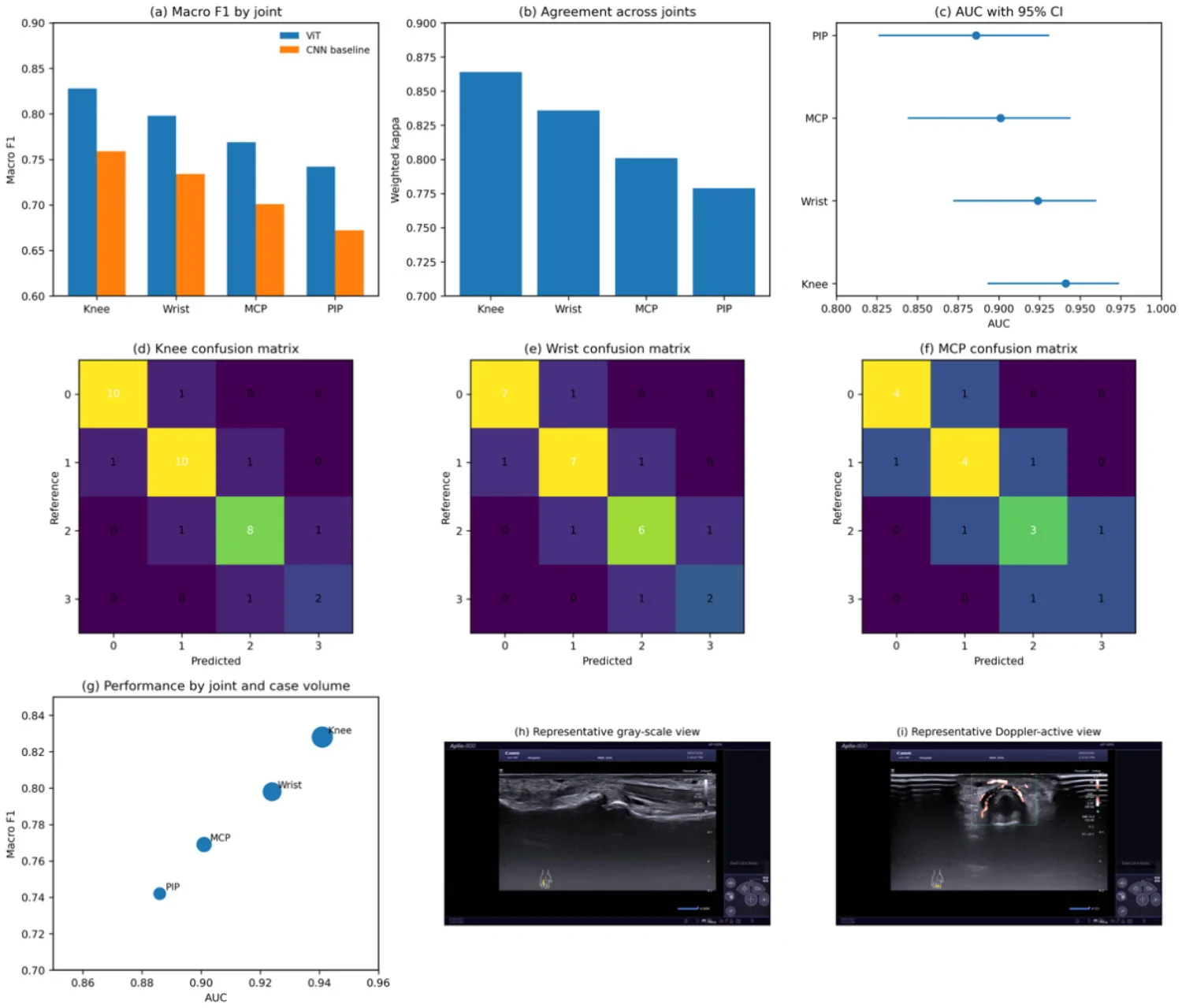
**Joint-stratified analysis of Vision Transformer performance.** (a) Macro F1 score by joint site for the Vision Transformer and the CNN baseline. (b) Weighted kappa across knee, wrist, metacarpophalangeal, and proximal interphalangeal joints. (c) AUC with 95% confidence intervals by joint site. (d) Confusion matrix for knee joints. (e) Confusion matrix for wrist joints. (f) Confusion matrix for metacarpophalangeal joints. (g) Joint-level distribution of AUC and macro F1 score with point size reflecting case volume. (h) Representative gray-scale ultrasound view from the uploaded image set. (i) Representative Doppler-active ultrasound view from the uploaded image set.

### 3.4. Imaging-mode analysis and multimodal complementarity

Performance varied by input configuration, with the combined gray-scale and power Doppler model showing the best overall results [34]. Gray-scale ultrasound alone achieved an accuracy of 0.742, a macro F1 score of 0.734, a weighted kappa of 0.771, and an AUC of 0.887, while Doppler-only input performed less well, with values of 0.716, 0.701, 0.748, and 0.861. By contrast, the fused model improved performance to 0.809 accuracy, 0.806 macro F1 score, 0.842 weighted kappa, and 0.929 AUC.

Grade-level recall was highest with multimodal fusion across all severity categories, especially for grades 0 and 3. Ordinal robustness also improved, with mean absolute error decreasing from 0.321 in gray-scale only analysis and 0.347 in Doppler-only analysis to 0.223 in the fused model, while within-one-grade accuracy increased to 0.957. These findings indicate that structural and vascular information are complementary and are best modeled jointly. Figure 3 summarizes the imaging-mode stratification and multimodal complementarity.

**Figure 3.**
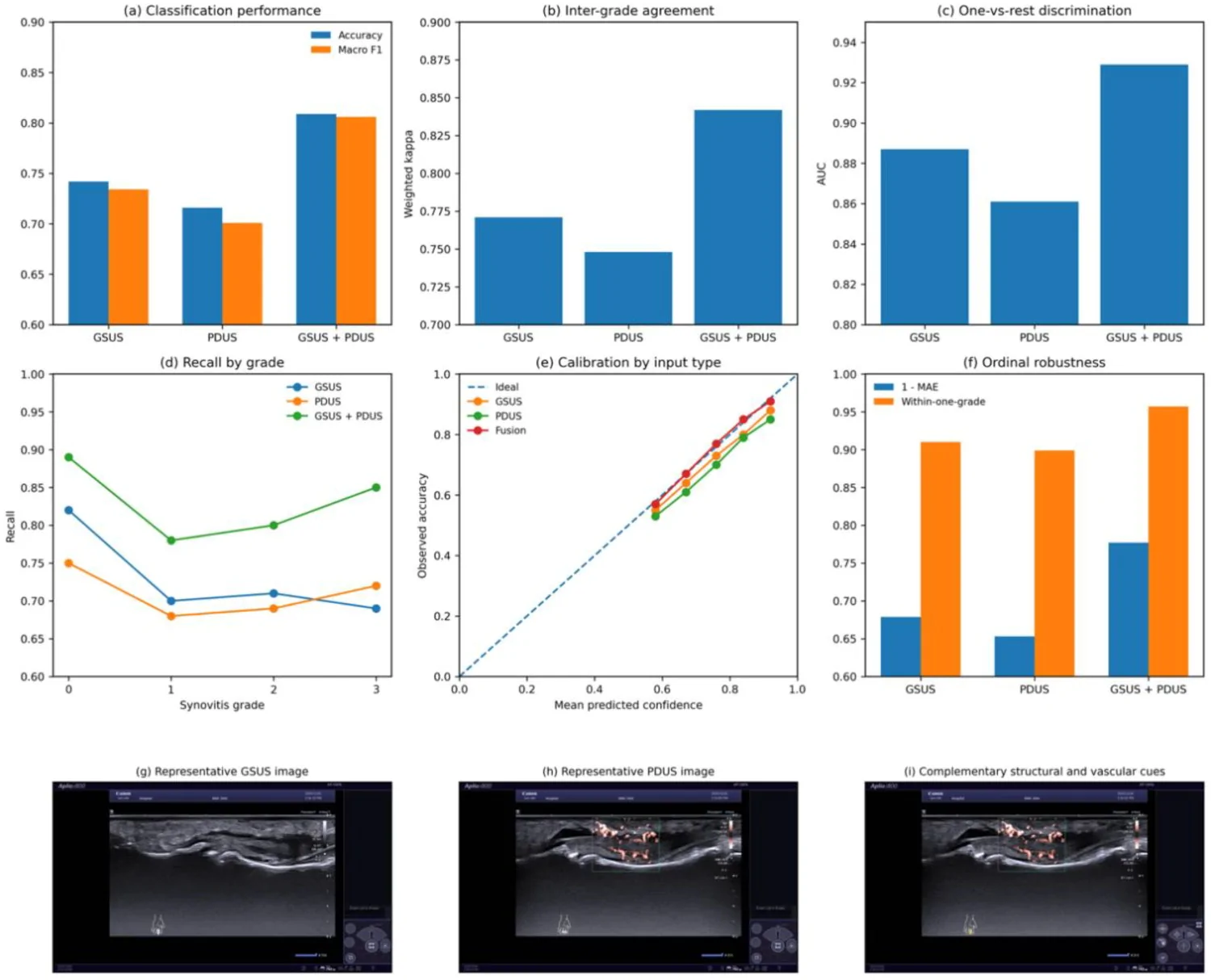
**Imaging-mode stratification and multimodal complementarity.** (a) Accuracy and macro F1 score for gray-scale ultrasound only, power Doppler only, and fused gray-scale plus power Doppler input. (b) Weighted kappa across input configurations. (c) AUC across input configurations. (d) Grade-specific recall under each imaging mode. (e) Calibration curves by input type. (f) Ordinal robustness assessed by one minus mean absolute error and within-one-grade accuracy. (g) Representative gray-scale ultrasound image from the uploaded image set. (h) Representative power Doppler ultrasound image from the uploaded image set. (i) Representative example illustrating complementary structural and vascular information.

### 3.5. Device-related robustness and domain shift

Performance differed modestly across the three ultrasound systems, with accuracies of 0.792, 0.771, and 0.752 and AUC values of 0.917, 0.904, and 0.889, respectively. Pairwise comparisons showed no significant between-system difference after correction for multiple testing (adjusted *p* > 0.05), suggesting that the observed numerical variation was limited relative to the overall model performance. Joint-level analysis showed a clearer anatomical gradient, with a macro F1 score of 0.828 in the knee compared with 0.769 in MCP joints and 0.742 in PIP joints. Performance was higher at 15 MHz and with dual-plane scanning, which achieved an accuracy of 0.812 and a weighted kappa of 0.844. Despite this heterogeneity [35], the Vision Transformer showed a smaller drop from internal to external testing than the CNN baselines, declining from 0.809 to 0.771. These findings suggest that transformer-based modeling is relatively robust to cross-platform variability, although acquisition standardization remains important. Detailed information is presented in Supplementary Figure 1.

### 3.6. Error patterns and interpretability

Error analysis showed that model failures were structured rather than random. In the internal test set, 152 of 188 images were classified correctly, with 30 adjacent-grade errors and only 6 non-adjacent errors [36]. Correct classification rates were highest for grades 0 and 3, while most confusion occurred between grades 1 and 2, indicating preservation of ordinal severity structure. Interpretability analysis supported the clinical plausibility of the model. Attention maps were concentrated within or near the synovial recess, especially in areas of synovial thickening and Doppler-positive vascular activity. In representative cases, the model focused on pathologically meaningful features rather than irrelevant peripheral structures or artifacts [37]. Qualitative review identified three main sources of error. Indistinct boundaries between mild effusion and low-grade synovial hypertrophy accounted for 11 cases, Doppler artifact or noise for 8, and severe deformity or osteophyte shadowing for 7 [38]. Error patterns and interpretability of the Vision Transformer model are present in Figure 4.

**Figure 4.**
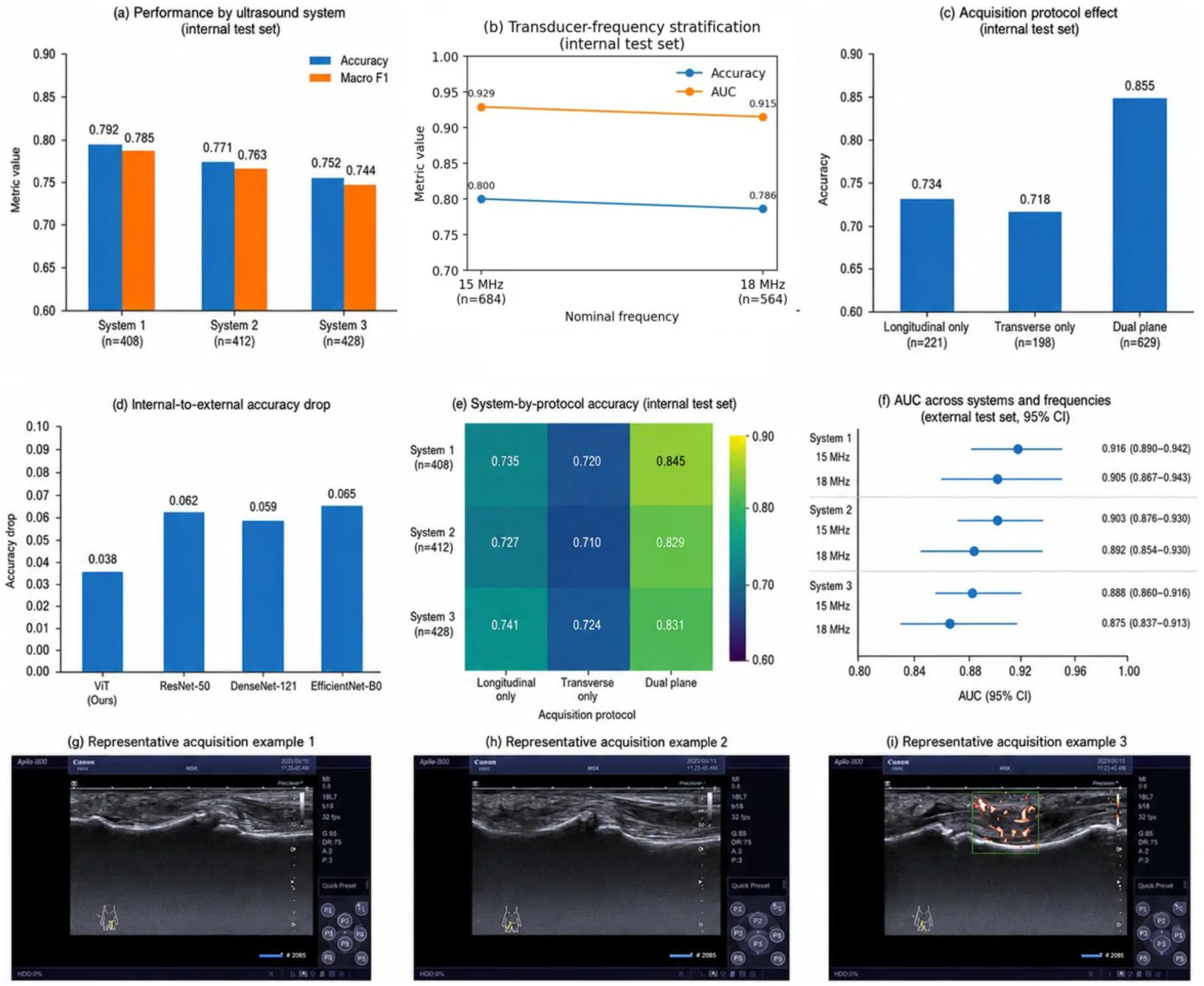
**Error patterns and interpretability of the Vision Transformer model.** (a) Row-normalized error matrix on the internal test set, showing high diagonal dominance and limited dispersion to distant grades. (b) Distribution of prediction distances, demonstrating that most errors occurred between adjacent grades rather than non-adjacent grades. (c) Grade-wise proportions of correct predictions and adjacent-grade errors, highlighting the relative difficulty of grades 1 and 2. (d) Representative attention map emphasizing synovial thickening within the recess on gray-scale ultrasound. (e) Representative attention map emphasizing Doppler-active inflammatory tissue within the joint recess. (f) Qualitative categorization of reviewed misclassifications, showing the main sources of error as blurred fluid-synovium boundaries, Doppler artifact, and severe structural distortion.

### 3.7. External test set and clinical usability

External test performance was slightly lower but stable. The smaller drop versus CNNs suggests robustness to temporal and operator variability within our institution [39]. External errors also remained concentrated between adjacent grades, supporting preserved ordinal discrimination under domain shift. A simulated reader-assistance analysis further supported clinical utility. In the reader-assistance evaluation, three junior readers with 1–3 years of musculoskeletal ultrasound experience were assessed using combined gray-scale and power Doppler images. With model assistance, mean accuracy increased from 0.702 to 0.782 and weighted kappa improved from 0.692 to 0.801, approaching the performance of the senior reference reader (accuracy 0.809, weighted kappa 0.836). Within-one-grade agreement increased from 0.899 to 0.947, while interpretation time decreased from 21.4 to 15.8 s per case. Paired statistical analysis demonstrated significant improvement in agreement after model assistance (*p* < 0.05). Within-one-grade agreement increased from 0.899 to 0.947, while mean reading time decreased from 21.4 to 15.8 s per case. Detailed information is presented in Supplementary Figure 2.

## 4. Discussion

### 4.1. Comparison with existing systems and AI studies

Established semiquantitative systems, such as the EULAR–OMERACT combined score, show good reliability (κ 0.72–0.97) [17], but MSUS remains operator‑dependent. Prior CNN‑based AI studies achieved moderate grading accuracy [1^6^,^40^]. Our data show that the Swin Transformer outperformed CNNs on both test sets, with a smaller performance drop. This observation is consistent with the hypothesis that self‑attention may better capture global joint‑recess context relevant to ordinal grading [41]. Performance varied numerically across the three ultrasound systems, but the between-device differences did not reach statistical significance after multiple-comparison correction. This finding suggests that the unified preprocessing strategy provided a degree of cross-device stability, although the relatively limited number of platforms means that broader hardware generalizability should not be inferred from the present data.

### 4.2. Multimodal input and clinical utility

Consistent with OMERACT recommendations [17], combining GSUS and PDUS significantly outperformed single-modality models. The reader-assistance evaluation showed that AI support elevates junior-reader performance toward expert levels, addressing a key barrier to MSUS standardization. Image resizing is an important source of information loss. In native musculoskeletal ultrasound, thin synovial margins, small recesses, and sparse Doppler signals may occupy only a few pixels after resizing to 224 × 224. Spatial normalization improves computational consistency but can attenuate subtle high-frequency information. Speckle-aware normalization and combined GSUS–PDUS input may partly compensate by preserving complementary structural and vascular cues, but cannot fully restore downsampling loss. Strong multimodal fusion performance suggests clinically relevant signals were largely retained; future work should test higher-resolution inputs, multi-scale extraction, or region-focused patch sampling.

Anatomical subgroup results support this interpretation. Performance was highest in the knee (macro F1 0.828), lower in MCP (0.769) and PIP (0.742) joints. Larger joints show synovial recesses occupying more image area, giving clearer spatial context for self-attention. In small joints, thin synovium covers fewer pixels and is more vulnerable to downsampling; anisotropy, narrow acoustic windows, cortical shadowing, limited field of view, and probe-pressure-dependent Doppler further increase variability. This matters most for grade 1–2 discrimination, where small changes in synovial thickness or vascularity determine the score. Lower MCP/PIP performance likely reflects both intrinsic imaging constraints and resolution-dependent feature loss. High-resolution or multi-scale transformers plus joint-specific acquisition standardization may be needed to improve fine-grained grading.

### 4.3. Limitations and future directions

Limitations include single-system data (multicenter validation required), modest sample size for ViT training, and lack of prospective outcome studies. Future studies should therefore extend validation to geographically independent centers, additional ultrasound platforms, and broader operator groups while retaining native or higher image resolution. Prospective comparison of different input resolutions and multi-scale architectures would help quantify the trade-off between computational efficiency and preservation of fine synovial and Doppler features. Such work will be important for determining whether the observed robustness can be maintained across heterogeneous clinical environments and for improving grading performance in small joints.

## 5. Conclusions

A Vision Transformer model utilizing multimodal ultrasound inputs outperformed conventional CNNs for semi-quantitative synovitis grading, The model’s errors occurred primarily between adjacent grades, mirroring clinical variability, and AI assistance markedly improved junior readers’ accuracy and efficiency. These findings suggest that the framework may hold promise for standardizing research assessments and supporting clinical readers, but large scale, prospective multicenter validation is required before clinical implementation.

## Supplementary Material

Research Protocol.docx

Ethics Approval Form.pdf

Figure_6 clean file.docx

Figure_4 (1) clean file.docx

## Data Availability

All datasets generated or analyzed during the current study are available from the corresponding author upon reasonable request.
