# Supplementary material for: Vision transformer-based ordinal model for semi-quantitative synovitis grading on musculoskeletal ultrasound: a retrospective study with internal and external validation
Source: Ann Med. 2026 Oct 1;58(1):2738281. doi: 10.1080/07853890.2026.2738281 (PMC13637757; doi:10.1080/07853890.2026.2738281)
Supplement: Supplementary file 1 [file IANN_A_2738281_SM9467.pdf]

天津医大二院医学伦理委员会

天津医科大学第二医院

医学伦理委员会快审批件

科审第（KY2026K274）号

项目名称	基于视觉转换器的肌骨超声滑膜炎分级		
项目来源	发表论文		
申请科室	超声科	项目负责人	刘欣

审查内容：

1、伦理审查申请表
2、研究方案 版本号 1.0；版本日期：2026.03.18
3、免知情同意申请书

主审委员名单及委员签名

委员姓名	职 务	审查意见	签 名	日 期
刘 毅	委员	☑同意 ☐作必要修正后同意 ☐不同意 ☐作必要修正后重审 ☐终止或暂停已批准的研究 ☐提交会审	刘毅	2026.4.1
唐 健	委员	☑同意 ☐作必要修正后同意 ☐不同意 ☐作必要修正后重审 ☐终止或暂停已批准的研究 ☐提交会审	[signature]	2026.4.1

评审结果：

☑同意开展项目
☐同意申报项目
☐作必要修正后同意
☐不同意
☐提交会审
☐终止或暂停已批准的研究

主任（副主任）委员签字：[signature]

医学伦理委员会（盖章）：天津医科大学第二医院医学伦理委员会

2026 年 4 月 1 日

扫描全能王
3亿人都在用的扫描App
