# Supplementary material for: Vision transformer-based ordinal model for semi-quantitative synovitis grading on musculoskeletal ultrasound: a retrospective study with internal and external validation
Source: Ann Med. 2026 Oct 1;58(1):2738281. doi: 10.1080/07853890.2026.2738281 (PMC13637757; doi:10.1080/07853890.2026.2738281)
Supplement: Supplementary file 2 [file IANN_A_2738281_SM9465.docx]

**Figure 4. Robustness across vendors, transducer frequencies, and acquisition protocols.**
(a) Accuracy and macro F1-score by device vendor. (b) Accuracy and AUC across transducer-frequency strata. (c) Accuracy according to acquisition protocol. (d) Internal-to-external accuracy drop across model architectures. (e) Heatmap of accuracy across vendor-by-protocol combinations. (f) AUC with 95% confidence intervals for vendor and frequency subgroups. (g) Representative acquisition example 1 from the uploaded image set. (h) Representative acquisition example 2 from the uploaded image set. (i) Representative acquisition example 3 from the uploaded image set.
