# Supplementary material for: Vision transformer-based ordinal model for semi-quantitative synovitis grading on musculoskeletal ultrasound: a retrospective study with internal and external validation
Source: Ann Med. 2026 Oct 1;58(1):2738281. doi: 10.1080/07853890.2026.2738281 (PMC13637757; doi:10.1080/07853890.2026.2738281)
Supplement: Supplementary file 3 [file IANN_A_2738281_SM9466.docx]

**Figure 6. External validation and clinical usability of the Vision Transformer model.** (a) Comparison of internal and external test accuracy across the Vision Transformer and CNN baseline architectures, demonstrating performance retention under independent-site evaluation. (b) Difference between internal and external accuracy for each model, representing the magnitude of performance degradation caused by dataset shift. (c) Confusion matrix of the Vision Transformer on the external test cohort, showing grade-specific classification patterns and the predominance of adjacent-grade errors. (d) Comparison of diagnostic performance between junior readers without model assistance, junior readers with AI assistance, and senior reference readers, including accuracy and weighted kappa. (e) Evaluation of workflow efficiency and grading consistency, including interpretation time per case and within-one-grade agreement under different reading conditions. (f) Decision-curve analysis comparing the clinical net benefit of AI-assisted reading and unaided junior interpretation across different threshold probabilities.
