# Supplementary material for: Vision transformer-based ordinal model for semi-quantitative synovitis grading on musculoskeletal ultrasound: a retrospective study with internal and external validation
Source: Ann Med. 2026 Oct 1;58(1):2738281. doi: 10.1080/07853890.2026.2738281 (PMC13637757; doi:10.1080/07853890.2026.2738281)
Supplement: Supplementary file 4 [file IANN_A_2738281_SM9468.docx]

**Research Protocol**

**I. Basic Protocol Information**

Protocol Title: Vision Transformer-Based Ordinal Model for Semi-Quantitative Synovitis Grading on Musculoskeletal Ultrasound

Protocol Version: 1.0

Version Date: May 10, 2026

Study Type: Retrospective Diagnostic Study

Study Period: April 2025 – April 2026

Study Institution: Department of Ultrasound, Second Hospital of Tianjin Medical University

Principal Investigators: Xin Liu, Jing Li

Corresponding Author: Jing Li [(jing0928@tmu.edu.cn)](mailto:(jing0928@tmu.edu.cn))

**II. Research Abstract**

Background: Synovitis is an important imaging feature of musculoskeletal disorders. Ultrasound is a commonly used assessment tool; however, grading remains operator-dependent and suffers from significant inter-observer variability.

Objective: To develop and validate a Vision Transformer-based model for semi-quantitative synovitis grading and to assess its clinical utility compared with conventional CNNs.

Methods: This retrospective study included 312 patients (624 joints, 1,248 ultrasound images), divided at the patient level into training, validation, internal test, and external test cohorts. A Swin Transformer-based ordinal grading model was developed and compared with CNN baselines through multi-dimensional evaluation.

Primary Endpoints: Accuracy, macro F1-score, weighted Kappa, and AUC.

**III. Study Objectives and Hypotheses**

Primary Objective: To develop and validate a Vision Transformer-based synovitis grading model.

Secondary Objectives: (1) Comparison with CNNs; (2) Evaluation of multimodal input benefits; (3) Assessment of cross-device generalizability; (4) Evaluation of AI assistance on junior reader performance.

Research Hypotheses: Vision Transformer outperforms conventional CNNs; multimodal input outperforms single-modality input; AI assistance improves reader agreement.

**IV. Methods**

**4.1 Study Participants**

Inclusion Criteria: (1) Age ≥ 18 years; (2) Symptomatic peripheral joints (MCP, PIP, knee, or wrist); (3) Standardized grayscale and power Doppler ultrasound images available; (4) Complete clinical records accessible.

Exclusion Criteria: (1) Poor image quality; (2) Postoperative deformity or joint replacement; (3) Artifacts obscuring the synovial recess; (4) Duplicate follow-up scans.

Sample Size: 312 patients (1,248 images), with ≥ 200 images per grade, satisfying deep learning heuristics.

**4.2 Data Source**

Three ultrasound systems were used: Mindray Resona A20, Aplio i800, and GE Fortis, all equipped with linear-array transducers (18/15 MHz).

**4.3 Reference Standard**

Synovitis was graded according to the EULAR-OMERACT semi-quantitative scoring system (0–3 scale). Two senior musculoskeletal sonographers independently annotated all images, with disagreements resolved by a third expert. Pre-adjudication inter-reader agreement was substantial, with a quadratic-weighted Cohen's kappa of 0.81.

**4.4 Model Development**

Architecture: Swin Transformer-Tiny with ordinal prediction head.

Loss Function: L = L_CE + 0.5 × L_ord, combining weighted cross-entropy with an ordinal consistency term.

Training Configuration: ImageNet pretraining; AdamW optimizer; initial learning rate of 1e-4; weight decay of 0.05; batch size of 32; early stopping triggered when validation loss failed to improve for 12 consecutive epochs.

Comparator Models: ResNet-50, DenseNet-121, and EfficientNet-B0.

**4.5 Reader-Assistance Evaluation**

Three junior musculoskeletal ultrasound readers (1–3 years of experience) participated. In the unassisted session, readers graded test cases without model input. After a 4-week washout period to minimize recall bias, the same readers re-evaluated the cases with access to model-generated grade predictions and confidence outputs. A senior sonographer (>8 years) provided the reference standard. All readers were blinded to expert labels, diagnoses, and model performance; case order was randomized.

Timeline: The unassisted session was conducted in the first week of March 2026, and the assisted session was conducted in the first week of April 2026.

**4.6 Outcome Measures**

Primary Outcomes: Accuracy, macro F1-score, weighted Kappa, and area under the ROC curve (AUC).

Secondary Outcomes: Mean absolute error, within-one-grade accuracy, per-class recall, and reading time per case.

**V. Ethics and Informed Consent**

Ethics Approval: The study protocol was reviewed and approved by the Ethics Committee of the Second Hospital of Tianjin Medical University (IRB Number: KY2026K274).

Informed Consent: The requirement for informed consent was waived by the ethics committee due to the retrospective design and the use of fully anonymized data.

Guiding Principle: This study was conducted in accordance with the ethical principles of the Declaration of Helsinki.

**VI. Study Timeline**

Data Collection: April 2025 – March 2026

Image Annotation: May 2025 – March 2026

Data Preprocessing and Dataset Partitioning: March 2026 – April 2026

Model Development and Validation: March 2026 – April 2026

Reader-Assistance Evaluation: March 2026 – April 2026 (unassisted: first week of March; assisted: first week of April, after a 4-week washout period)

Data Analysis and Manuscript Finalization: April 2026

**VII. Data Management**

All images were anonymized prior to analysis, with patient identifiers, machine annotations, and non-imaging borders removed. Data were stored on a secure hospital server accessible only to the research team. All datasets generated or analyzed during the current study are available from the corresponding author upon reasonable request.

**VIII. Other Declarations**

Funding: The funder had no role in study design, data collection, analysis, interpretation, or manuscript preparation.

Conflicts of Interest: All authors declare no conflicts of interest related to this study.
